# Power to Detect Risk Alleles Using Genome-Wide Tag SNP Panels

**DOI:** 10.1371/journal.pgen.0030170

**Published:** 2007-10-05

**Authors:** Michael A Eberle, Pauline C Ng, Kenneth Kuhn, Lixin Zhou, Daniel A Peiffer, Luana Galver, Karine A Viaud-Martinez, Cynthia Taylor Lawley, Kevin L Gunderson, Richard Shen, Sarah S Murray

**Affiliations:** Illumina, San Diego, California, United States of America; University of Liège, Belgium

## Abstract

Advances in high-throughput genotyping and the International HapMap Project have enabled association studies at the whole-genome level. We have constructed whole-genome genotyping panels of over 550,000 (HumanHap550) and 650,000 (HumanHap650Y) SNP loci by choosing tag SNPs from all populations genotyped by the International HapMap Project. These panels also contain additional SNP content in regions that have historically been overrepresented in diseases, such as nonsynonymous sites, the MHC region, copy number variant regions and mitochondrial DNA. We estimate that the tag SNP loci in these panels cover the majority of all common variation in the genome as measured by coverage of both all common HapMap SNPs and an independent set of SNPs derived from complete resequencing of genes obtained from SeattleSNPs. We also estimate that, given a sample size of 1,000 cases and 1,000 controls, these panels have the power to detect single disease loci of moderate risk (λ ∼ 1.8–2.0). Relative risks as low as λ ∼ 1.1–1.3 can be detected using 10,000 cases and 10,000 controls depending on the sample population and disease model. If multiple loci are involved, the power increases significantly to detect at least one locus such that relative risks 20%–35% lower can be detected with 80% power if between two and four independent loci are involved. Although our SNP selection was based on HapMap data, which is a subset of all common SNPs, these panels effectively capture the majority of all common variation and provide high power to detect risk alleles that are not represented in the HapMap data.

## Author Summary

Advances in high-throughput genotyping technology and the International HapMap Project have enabled genetic association studies at the whole-genome level. Our paper describes two genome-wide SNP panels that contain tag SNPs derived from the International HapMap Project. Tag SNPs are proxies for groups of highly correlated SNPs. Information can be captured for the entire group of correlated SNPs by genotyping only one representative SNP, the tag SNP. These whole-genome SNP panels also contain additional content thought to be overrepresented in disease, such as amino acid–changing nonsynonymous SNPs and mitochondrial SNPs. We show that these panels cover the genome with very high efficiency as measured by coverage of all HapMap SNPs and a set of SNPs derived from completely resequenced genes from the Seattle SNPs database. We also show that these panels have high power to detect disease risk alleles for both HapMap and non-HapMap SNPs. In complex disease where multiple risk alleles are believed to be involved, we show that the ability to detect at least one risk allele with the tag SNP panels is also high.

## Introduction

Researchers have used several approaches to identify genetic variation that predispose individuals to common diseases. For Mendelian disease traits, the method of choice has been linkage analysis in families [1]. For many complex disease traits where many alleles contribute to the trait, the effect of any single gene or locus may be relatively small and difficult to detect by linkage analysis. A more sensitive approach to identifying risk alleles with smaller gene effects is to employ a case-control association study in which allelic markers, such as SNPs, are used to find regions of the genome enriched (or depleted) in a particular risk allele or haplotype between the cases and controls. This study design can provide more power to detect relatively small gene effects [1]. Historically, association studies have employed markers in candidate genes; unfortunately this approach requires a priori knowledge about which genes to choose. A more comprehensive and agnostic approach is to employ markers encompassing the entire genome. Whole-genome association studies survey common genetic variation by probing a dense set of SNPs across the genome. Because whole-genome studies allow researchers to identify genes not previously known to be involved with disease etiology and may be more sensitive for detecting the multiple small gene effects often found in complex disease traits, they may be the most appropriate method to identify variants that predispose individuals to common diseases.

Whole-genome association studies require a dense map of hundreds of thousands of SNPs across the genome and sufficiently large sample sizes to provide power to detect relatively small gene effects [2,3], but knowledge of the underlying LD (LD) structure can be used to minimize the number of genotyped SNPs [4,5]. The International HapMap Project [6] has provided a densely mapped and validated set of SNP loci genotyped in four populations that can be used to select these SNPs. The complete HapMap dataset (release 21) contains over 2 million common SNPs (minor allele frequency [MAF] ≥0.05) in each population studied (Utah residents with ancestry from Northern and Western Europe [CEU], Han Chinese/Japanese in Tokyo, Japan [CHB + JPT], and Yoruba in Ibadan, Nigeria [YRI]). One of the many results shown in the HapMap data is that LD is often discontinuous, appearing as “block-like” structures [7,8], and a typical SNP is highly correlated with many of its neighbors in any given population. This correlation can be used to build highly efficient whole-genome genotyping panels by choosing tag SNPs that serve as proxies for many other highly correlated neighboring SNPs [9]. A tag SNP approach drastically reduces the number of genotyped loci while producing more information content than a large set of randomly-chosen loci [8,10].

We have recently developed a whole-genome genotyping assay [11,12] that has the capability of genotyping hundreds of thousands of markers, enabling whole-genome association studies on a single microarray. Using this assay, we have constructed whole-genome genotyping panels of over 550,000 (HumanHap550) and 650,000 (HumanHap650Y) SNP loci by choosing tag SNPs from all populations in the International HapMap Project. We estimate that these tag SNP loci cover the majority of all common variation in the genome and show that most association studies using these panels should detect genetic risk factors even for complex traits where each risk allele only confers a moderate risk.

## Results

### Content Selection

We constructed the HumanHap550 panel, a whole-genome genotyping panel of 555,352 SNPs, to effectively tag CEU (European) and CHB + JPT (Asian) sample populations (Table 1). A majority of the SNPs were selected by tagging the more than 2 million common HapMap SNPs, but the panel also includes variation types that have been found to be overrepresented in diseases such as nonsynonymous SNPs [1], SNPs in the MHC region [13], SNPs in commonly reported CNV regions [14], and mitochondrial SNPs [15]. Because individuals with African ancestry have distinct and lower levels of LD compared to those with European or Asian ancestry [16], we added another 100,000 common YRI (African) tag SNPs to increase coverage of the YRI samples for the HumanHap650Y panel. In this publication, genome coverage and power are calculated using the HumanHap550 panel for CEU and CHB + JPT samples and the HumanHap650Y panel for YRI samples. Coverage calculation has been previously described for a subset of ∼314,000 SNPs from these panels (the HumanHap300 panel) [17,18].

**Table 1:**
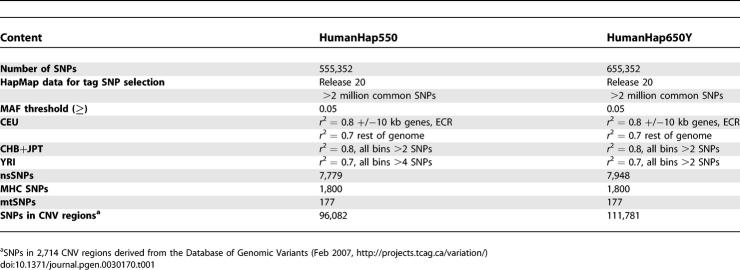
HumanHap550 and HumanHap650Y Coverage

### Genome Coverage Estimations

We assessed how well the whole-genome genotyping panels capture all variation in the human genome. To do this, we measured how well all variation is captured by at least one SNP on the array at various levels of LD as measured by *r* ^2^ [19,20]. Since all variation in the genome is currently not known, one can use proxies for all variation to estimate genomic coverage. These include SNPs genotyped in the International HapMap Project [6], the HapMap ENCODE resequencing and genotyping project [21], and other complete resequencing data such as the SeattleSNPs Program for Genomic Applications (PGA) data (http://pga.gs.washington.edu/). Genomic coverage was estimated from HapMap release 21 and SeattleSNPs PGA genotype data. Because the PGA data contains 68 genes whose variants were publicly released after the HapMap SNPs had been selected, and HumanHap550 and HumanHap650Y tag SNPs were derived from the HapMap, this dataset provides a relatively unbiased estimate of genomic coverage beyond HapMap. Since most of the SNPs genotyped in the HapMap ENCODE regions were part of the HapMap release 16c data, estimates of genomic coverage in ENCODE regions is not an independent assessment of genomic coverage. Coverage of ENCODE regions was very similar to coverage of the entire HapMap data and is not shown.

**Figure 1:**
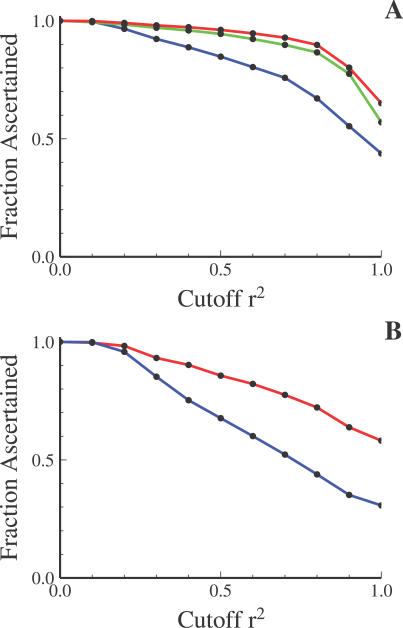
Coverage of the HapMap (A) or SeattleSNPs (B) Datasets As Measured by the Proportion of Common Variation in Each Dataset That Is Captured by a SNP in Either HumanHap550 (CEU; CHB + JPT) or HumanHap650Y (YRI) at Various *r* ^2^ Thresholds Lines show coverage for the CEU (red), CHB + JPT (green), and YRI (blue) populations.

Coverage of common variation (MAF ≥ 0.05) was estimated from the HapMap data (release 21) for each population (CEU, CHB + JPT, YRI). To calculate coverage, the maximum pairwise *r* ^2^ value [19,20] between each common HapMap release 21 SNP and a SNP in either HumanHap550 or HumanHap650Y was determined. The cumulative proportion of HapMap release 21 loci captured by HumanHap550 and HumanHap650Y is shown in Figure 1. The mean maximum *r* ^2^ was 0.93 and 0.91 between HumanHap550 SNPs and common HapMap SNPs in the CEU and CHB + JPT samples, respectively, and 0.81 for HumanHap650Y SNPs in the YRI samples. Using a strict *r* ^2^ threshold of 0.8, HumanHap550 captures 90% and 87% of the common HapMap SNPs in the CEU and CHB + JPT samples, respectively, and the HumanHap650Y captures 67% of common HapMap SNPs in YRI samples. Using a less-stringent *r* ^2^ threshold (0.5), the HumanHap550 panel captures 96% and 95% of the common HapMap variants in the CEU and CHB + JPT samples, respectively, and the HumanHap650Y captures 85% of common variants in YRI samples. Because both SNP selection and genomic coverage of the HumanHap550 panel was based on the same HapMap data, these estimates are likely to be an overestimate of true genomic coverage.

Although the International HapMap Project genotyped over 5.8 million SNPs in four populations, these SNPs do not necessarily represent all genomic variation due to biases in SNP discovery and genotyping. Furthermore, as mentioned previously, there is a slight upward bias for our estimate of genomic coverage because the SNP selection used for these panels was based on the samples and markers in the HapMap project. An unbiased estimate of the coverage of these panels can be obtained from regions that are fully sequenced independently from the HapMap project. Regions sequenced before the HapMap project may be overrepresented in the HapMap data because more complete knowledge existed for these regions when SNPs were selected for the HapMap project. The SeattleSNPs Program for Genomic Applications (PGA; http://pga.gs.washington.edu/) has resequenced 68 genes (as of 29 September 2006) related to inflammatory response in 23 and 24 individuals in common with the HapMap CEU and YRI samples, respectively. The PGA's earliest SNP entries into dbSNP are build 125 for these 68 genes, after when SNPs from the HapMap Project were selected (http://www.hapmap.org/) [6]. The HumanHap550 panel has a mean maximum *r* ^2^ of 0.84 and captures 72% of common variation in the CEU population (*r* ^2^ = 0.8), and HumanHap650Y has a mean maxiumum *r* ^2^ of 0.68 and captures 45% of the common variation in the YRI population (*r* ^2^ = 0.8), respectively (Figure 1B). These values are 18% and 22% less than what is seen in the HapMap data for the CEU and YRI samples, respectively. It is possible that the 68 genes in the PGA dataset may not accurately reflect all variation in the genome since these are candidate genes for inflammatory response and may be undergoing selection or have atypical patterns of natural variation. To test if these genes are undergoing selection, we calculated the average Tajima's D statistic for the 68 genes and found that they are close to neutrality and not significantly different from all genes for which data is available (Table S1).

### Power for a Single Risk Allele

Quantifying coverage according to *r* ^2^ does not fully address the probability that a study will detect risk alleles in genotype–phenotype association studies. Because the sample size required to maintain the same power is inversely proportional to *r* ^2^ [3,22,23], LD coverage allows us to estimate the reduction in power that will occur by not genotyping every SNP directly. Other factors are more important than *r* ^2^ for the power of an association study (i.e., the probability that a risk allele will be observed at significantly higher frequencies in the cases versus controls), such as the risk conferred by the allele and the frequency of the disease. In addition, the frequency of the risk allele is a very important factor in how much power an association study will have to detect the risk allele because low frequency risk alleles are much more difficult to detect than high frequency risk alleles. As long as the genotyped SNPs have some correlation with the risk alleles, increasing the sample size of a study can overcome all these factors so that a study can be sufficiently powered with a large enough sample size. Selecting SNPs in a way that maximizes the coverage of all SNPs through LD allows us to maximize our power for a given sample size.

### Power to Detect a Single HapMap Risk Allele

For a model where there exists a single risk allele, we estimated the total power to detect a single risk allele represented by the HapMap data where each SNP is equally likely to be the risk allele. For each SNP in the HapMap data, we combined the frequency and maximum *r* ^2^ with a nearby SNP on the HumanHap550 or HumanHap650Y to estimate the power to detect that SNP if it is a risk allele for various sample sizes and disease models. For each risk allele frequency, sample size, and disease model, the power was estimated from 10,000 simulated case-control datasets (see Methods). Then, under the assumption that each SNP in the HapMap dataset is equally likely to be the risk allele, we calculated the total power, *P_T_*, to detect a risk allele represented in the HapMap data for a 5% false-positive rate after applying a Bonferroni correction to account for multiple testing (see Methods).

**Figure 2:**
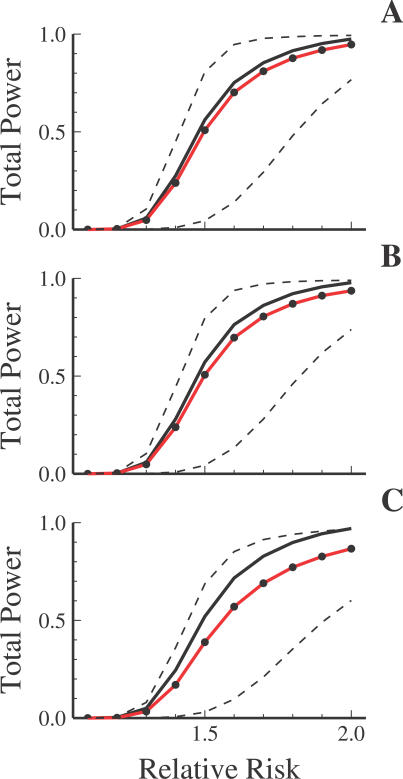
Total Power to Detect a Single Common Risk Allele in the HapMap Data Using HumanHap550 for CEU (A) and CHB + JPT (B) and HumanHap650Y for YRI (C) in a Study Size of 1,000 cases and 1,000 Controls under a Multiplicative Disease Model Power is calculated for a 5% false discovery rate after a Bonferroni correction for multiple testing (red line). The solid black line represents the power to detect a risk allele if all the common HapMap SNPs in each respective population are genotyped using the same significance cutoff. Also shown are the powers for different frequency ranges as dashed lines, where the lower line indicates the power for risk allele frequencies from 5%–10% and the upper line indicates the power for risk allele frequencies from 40%–50%.

Under the multiplicative disease model, an individual's baseline risk of having the disease or phenotype is multiplied by λ if they carry one copy of the risk allele and λ^2^ if they carry two copies of the risk allele. Figure 2 (red line) shows the total power to detect an association using HumanHap550 (CEU and CHB + JPT) and HumanHap650Y (YRI) for a study design with 1,000 cases and 1,000 controls for different relative risks, λ. For the CEU and CHB + JPT samples, the power drops below 80% when the relative risk is less than ∼1.69 under the multiplicative model, whereas for the YRI samples the power drops below 80% when the relative risk is less than ∼1.85 (Figure 2).

We have also performed the power analysis for an additive model where an individual's baseline risk is multiplied by λ if they carry one copy of the risk allele and 2λ if they carry two copies of the risk allele (see Figure S1). Under the additive model the power drops below 80% when the relative risk is less than ∼1.61 in the CEU and CHB + JPT samples and ∼1.80 in the YRI samples (Figure S2).

**Figure 3:**
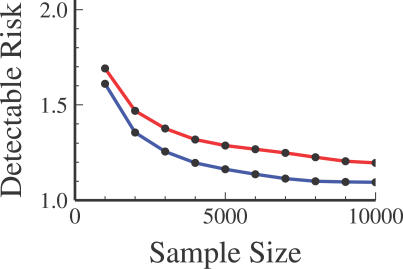
The Minimum Risk Detectable at 80% Power with *p* ≤ 0.05 after a Bonferroni Correction for Multiple Testing (χ^2^ ≥ 28.5687) for Various Sample Sizes under Multiplicative (Red) and Additive (Blue) Models Power is calculated as the ability to detect a single common risk allele in the HapMap data in a whole genome association study within CEU samples.

These simulations were based on sample sizes of 1,000 cases and 1,000 controls but a lower relative risk can be detected by increasing the study size. To illustrate this, we also estimated power in a variety of study sample sizes to examine the minimum relative risk detectable at 80% power under the multiplicative and additive models for larger sample sizes (Figure 3 [CEU] and Figure S2 [CHB + JPT and YRI]). Under the multiplicative model, disease variants with relative risks as low as ∼1.20 for the CEU and CHB + JPT samples and ∼1.24 for the YRI samples can be detected with 80% power with 10,000 cases and 10,000 controls (Figure S2; Tables S2 and S4). Under the additive model, diseases variants with relative risks as low as ∼1.06 for the CEU and CHB + JPT samples and ∼1.12 for the YRI samples can be detected with 80% power with 10,000 cases and 10,000 controls (Figure S2; Tables S3 and S5).

These power estimates are strongly influenced by the frequency spectra of the SNPs in the HapMap data. While the power is relatively low to detect a single risk allele if the minor allele frequency is less than 0.10, the power is significantly greater for risk alleles with minor allele frequencies greater than 0.40 (dashed lines in Figure 2). For example, under the multiplicative model, relative risks as low as 1.50–1.57 can be detected in 1,000 cases and 1,000 controls if the minor allele frequency is greater than 0.40, but when the minor allele frequency is less than 0.10, the risk allele can only be detected at this sample size if the relative risk is greater than 2. Unknown factors such as the risk allele frequency may significantly increase the power to detect a particular disease even though the above estimates indicate that a study is underpowered. Conversely, even a supposedly well-powered study may have little ability to detect associations if the risk allele frequencies are low, unless they confer a very large risk.

### Power to Detect a Single Non-HapMap Risk Allele

**Figure 4:**
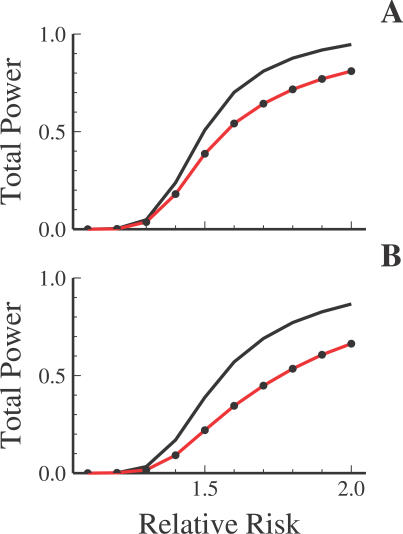
Total Power to Detect a Single Common Risk Allele outside of the HapMap Data Using 1,000 cases and 1,000 Controls under a Multiplicative Model The power is estimated using the common SNPs in 68 resequenced SeatleSNPs genes that were not characterized in the HapMap Project for (A) 23 CEU samples using HumanHap550 and (B) 24 YRI samples using HumanHap650Y. Power is calculated for a 5% false discovery rate after a Bonferroni correction for multiple testing (red line). The solid black line shows the power to detect a common risk allele in the HapMap data (i.e., solid red lines in Figure 2).

Since there are ∼7.1 million common (MAF ≥ 0.05) SNPs in the genome [24] and 2.0 million, 2.2 million, and 2.5 million common SNPs have been genotyped by the HapMap Project in the CHB + JPT, CEU, and YRI populations, respectively, approximately 70% of the common risk alleles are not contained in the HapMap data. For SNPs that are not contained in the HapMap data, the power will be lower than shown in Figures 2 and 3 because the tag SNPs selected for the HumanHap550 and HumanHap650Y were chosen specifically to maximize coverage of the HapMap SNPs. To explore how the HumanHap550 and HumanHap650Y will perform for the non-HapMap SNPs, we estimated the coverage of non-HapMap SNPs using the SNPs found in 68 genes that were completely resequenced by SeattleSNPs after the release of the HapMap data. It is important to note that for this analysis we excluded all SNPs that are in the HapMap dataset to provide an unbiased estimate of the power for SNPs outside of HapMap. Following the same procedure as outlined above, we estimated the power to detect the common SNPs (MAF ≥ 0.05) that are not in the HapMap data (Figure 4 multiplicative model; Figure S3 additive model). In 1,000 cases and 1,000 controls, the minimum relative risks that we can detect at 80% power is ∼14%–22% higher for non-HapMap SNPs compared with our estimates for just the HapMap data (Tables S2–S5). In 10,000 cases and 10,000 controls, the minimum relative risks that we can detect at 80% power is ∼6%–11% higher for non-HapMap SNPs compared with our estimates for just the HapMap data (Tables S2–S5). The SeattleSNPs dataset represents a small number of SNPs (668 for non-HapMap SNPs for CEU and 1,429 for YRI), so even though these genes represent over ∼2.4 Mb of the human genome, these are rough estimates and more extensive data will help refine these numbers. Overall, these estimates of power are very promising for studies that wish to detect risk alleles both within and outside of the HapMap SNPs.

### Genome-Wide Power Risk Loci

The total power to detect a common risk allele in an association study is a weighted average of the power to detect a HapMap risk allele and the power to detect a non-HapMap risk allele where the weights are the relative fraction of SNPs within and outside of the HapMap data (see Methods). We have combined our power calculations to estimate the genome-wide power for a variety of disease models and study designs (Tables S2–S5). In 1,000 cases and 1,000 controls, the minimum relative risks that we can detect at 80% power is ∼10%–18% higher genome wide compared with our estimates for just the HapMap data. In 10,000 cases and 10,000 controls the minimum relative risks that we can detect at 80% power is ∼4%–8% higher genome-wide compared with our estimates for just the HapMap data (Tables S2–S5).

### Power for Multiple Loci

The power calculations shown above were done assuming that a single, common risk allele is represented in the genome. For most complex traits with a significant genetic component, multiple risk alleles and loci are likely to be involved [25]; therefore, the power estimates calculated under the assumption that there is only one risk allele may be overly conservative. Risk alleles have been detected in multiple genes for many diseases [26–33].

**Figure 5:**
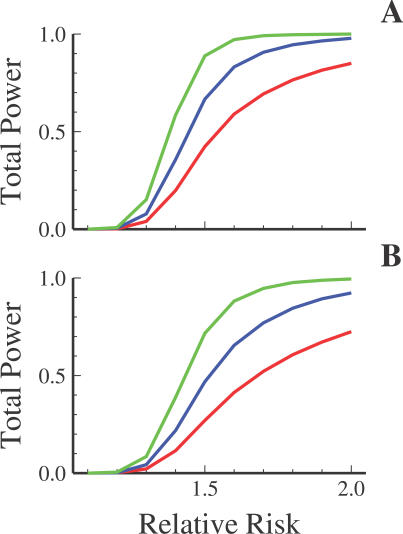
Power to Detect At Least One Risk Allele in CEU (HumanHap550) and YRI (HumanHap650Y) in 1,000 Cases and 1,000 Controls under a Multiplicative Model Curves represent the cases where there is a single risk locus (red), two independent loci (blue), or four independent loci (green). The red lines are the single risk loci as shown in Figure 4. Under the multiple loci cases, the relative risk represents the minimum risk for all risk alleles and the corresponding power represents a lower bound on the power.

While the most desirable case power-wise is to have a single high-risk allele that explains the entire genetic component of a disease, the presence of multiple risk loci will improve the power for association studies—compared with a single loci of the same frequency and risk—if the loci are segregating independently and noninteractively in the population. For example, if there are two risk alleles segregating independently, and one confers a multiplicative relative risk of λ = 1.5 and the other λ = 1.6, the genome-wide power to detect each SNP in 1,000 cases and 1,000 controls is 0.42 and 0.59, respectively (Table S2). However, the power to detect at least one of the two risk loci is one minus the probability that we do not detect either locus (0.76), greatly improving the power to detect any particular single locus. To further illustrate how the power improves when multiple loci are involved, we started from the whole-genome estimates and examined the case where a phenotype has genetic risk factors at two or four unlinked loci (see Methods). If two unlinked loci are involved, studies with the same sample sizes and power may be able to detect smaller gene effects (Figure 5). As shown in Tables 2 and 3, at 80% power, the minimum relative risk to detect at least one of the two risk alleles can be as much as 17% smaller for the multiplicative model and 23% for the additive model. If four unlinked loci are involved, even smaller gene effects may be detected at 80% power given the same sample size. The minimum relative risk detectable with 80% power can be as much as 23% smaller for the multiplicative model and 36% for the additive model when four unlinked loci are involved (Tables 2 and 3). These values require that each risk allele confers at least the same relative risk or greater and thus can only be considered rough estimates. Even so, the presence of multiple unlinked risk alleles will greatly improve the power of association studies.

**Table 2:**
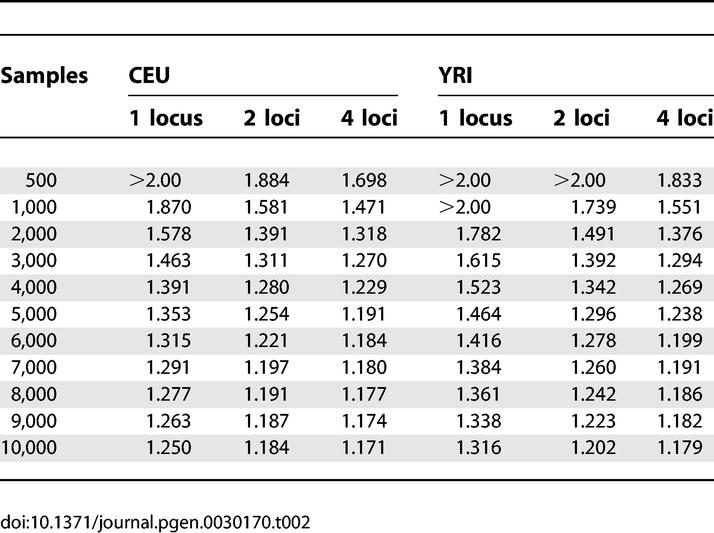
Minimum Risk Detectable at a *p-*Value ≤ 0.05 after a Bonferroni Correction for Multiple Testing (χ^2^ ≥ 28.5687 for CEU and χ^2^ ≥ 28.8976 for YRI) with 80% Power under the Multiplicative Model for Various Sample Sizes and Number of Unlinked Disease Loci

**Table 3:**
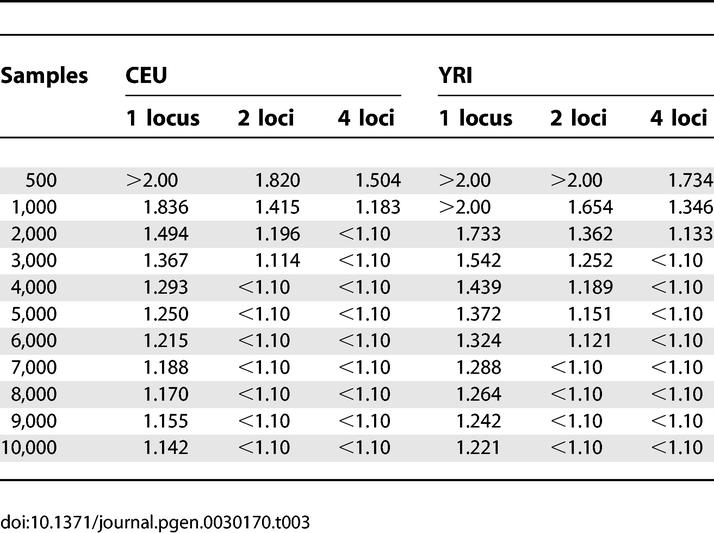
Minimum Risk Detectable at a *p-*Value ≤ 0.05 after a Bonferroni Correction for Multiple Testing (χ^2^ ≥ 28.5687 for CEU and χ^2^ ≥ 28.8976 for YRI) with 80% Power under the Additive Model for Various Sample Sizes and Number of Unlinked Disease Loci

## Discussion

Just as high-throughput sequencing and the human genome project have greatly advanced the study of genomics [34], so have advances in high-throughput genotyping and the HapMap project enabled association studies at a whole-genome level. Using data from the International HapMap Project [6], we have constructed genotyping panels for whole-genome association studies. These panels are invaluable tools for discovering etiologic variants of complex diseases. We have shown that, given a sample size of 1,000 cases and 1,000 controls, these panels have the power to detect single disease loci of moderate risk (λ ∼1.8–2.0). Relative risks as low as λ ∼1.2–1.3 can be detected using 10,000 cases and 10,000 controls, depending on the sample population and disease model. If multiple loci are involved, the power increases significantly such that relative risks 20%–35% lower can be detected with 80% power if between two and four independent loci are involved. Although our SNP selection was based on HapMap data, which is a subset of all common SNPs, these panels effectively capture the majority of all common variation and provide high power to detect risk alleles that are not represented in the HapMap. Additionally, we have focused on common (MAF ≥ 0.05) variants in this study, and it should be stressed that because there are many more rare variants in the population, there will likely be many more rare risk alleles. The power for these rare risk alleles will be much lower than the average power that we have used (e.g., dashed lines in Figure 2), and discovering these low-frequency risk alleles will likely require resequencing efforts.

Using the known relationship between sample size and *r* ^2^ [3,22], we estimated the probability of having a positive outcome under various scenarios of study size and different disease models (i.e., risks). Recently, this relationship between *r* ^2^ and sample size has been questioned [23]. It is true that if tag SNPs are selected using small sample sizes, the LD between SNPs will be upwardly biased compared with larger datasets but this bias will only be large if the training sample is small and/or the LD criteria used to select the tag SNPs is low. When tag SNPs are selected using high-LD criteria (e.g., *r* ^2^ > 0.5 ) and sample sizes are greater than ∼50 samples, this bias is minimal (e.g., Figure 1 of [23]). Because our tag SNPs are selected using a stringent *r* ^2^ criteria (*r* ^2^ > 0.8) and more than 50 individuals were genotyped in each of the CEU, CHB + JPT, and YRI populations, the bias in LD will be small for these tag SNPs [35].

One method to increase power in a whole-genome association study is to use imputation methods to infer genotypes for unobserved markers [30,36,37]. This method relies on phased genotype data from a known set of genotyped markers, i.e., the HapMap. The reliability of the imputed genotyped data is dependent on how well correlated the observed (genotyped) SNPs are to the unobserved (imputed) genotyped SNPs. Therefore, whole-genome genotyping panels selected using tag SNPs would likely provide more reliably imputed genotypes as the same number of non-tag SNPs. Reliability of the inferred genotypes is also dependent on the study population being similar to the HapMap population being used to impute genotypes.

The use of multi-marker haplotypes would also increase power in a whole-genome association study by providing greater specificity for detecting the actual risk allele [38–41]. In the extreme example where allelic heterogeneity exists and the disease variant occurs on two different haplotypes, where each haplotype does not contain any alleles in common (i.e., AB versus ab), the association would be missed using single-marker analysis, but could be detected if a haplotype analysis was used. The power estimated in this paper used single-marker analysis only, and therefore power could be improved beyond what is presented here if multi-marker haplotypes are used.

Since we made the assumption that risk alleles exist as either a single noninteractive SNP or a couple of unlinked loci, it is important to understand how our results would be affected if some of the risk alleles are in LD with one another. If multiple risk alleles are in LD with one another then they could mask the signal of each other and be much more difficult to detect in simple association studies. An extreme example of how one risk allele may mask another would occur is if two risk alleles (both of which confer the same risk) were in perfect LD such that every chromosome contained one of the risk alleles but never contained both (i.e., *D*′ = −1). In this example, the genetic risk factors would be impossible to detect using simple association studies. While this scenario may occur, it seems unlikely that this would be any more prevalent than a scenario where the risk alleles are correlated in such a way that they increase the probability of detection; e.g., both risk alleles always occur on the same chromosome (i.e., *D*′ = 1). Thus, our results probably represent the average scenario and some association studies will have more power and some will have less power, due to LD between risk alleles. A more difficult example would occur when there are epistatic interactions between loci that mask the overall gene effect signal that we would expect to get from each marker independently. Searching for these types of interactions will require at the very least multi-marker analyses. The power calculations presented here do not cover epistatic disease models and our results would substantially overestimate the power under these disease models.

Complex diseases as a group vary across of range of genetic risks, environmental factors, and other complexities. At one extreme are the simpler diseases that contain relatively strong genetic components, such as Crohn Disease (sibling relative risk, λs = 17–35 [42,43]) and Type I Diabetes (λs = 15 [44]). Isolating the high-risk genetic components for diseases similar to these may not require as many markers or samples because even SNPs that are loosely correlated with markers of these risks will be expected to show association. Conversely, diseases towards the other end of the spectrum of complexity, such as Type 2 Diabetes Mellitus (λs = 3 [45]), where many risk alleles, each with relatively small gene effects, are expected to occur, will require denser marker sets and larger samples sizes to detect an association [46]. To detect risk alleles, a key component is the design of genome-wide association studies. For example, in choosing patient samples, case subjects with specific disease subphenotypes are collected to decrease phenotypic heterogeneity and appropriate controls are used to eliminate false positives from population stratification [31,47] . A second key component to genome-wide association studies is the marker set. To address this, we have designed the HumanHap550 and HumanHap650Y whole-genome genotyping panels to maximize the likelihood of detecting etiologic variants. By selecting tag SNPs, we reduced the amount of required genotyping of more than 2 million common HapMap SNPs to ∼550,000–650,000 while retaining most of the power (e.g., Figure 2). Furthermore, to increase the chance of detecting etiologic variants, we have selected SNPs in gene regions and regions that have historically been overrepresented in disease, such as copy-number variation regions and mitochondrial DNA. Already, the success of genome-wide association studies are coming to bear and candidate risk alleles have been discovered for several disorders [26–33,47–56]. In summary, whole-genome tag SNP panels, such as the HumanHap550 and HumanHap650Y panels, should greatly aid in our understanding of how genetic variation affects both human health and disease.

## Methods

### Public data used.

HapMap release 16c, 20, and 21 data were downloaded from http://www.hapmap.org/. The filtered, nonredundant genotypes were used for all analyses. SeattleSNPs data were downloaded on 29 September 2006 from http://pga.gs.washington.edu/. SeattleSNPs sequenced 23 CEU and 24 YRI unrelated individuals from the HapMap samples. Tajima's *D* values for the SeattleSNPs genes were taken directly from the SeattleSNPs website. The 18,411 RefSeq genes and their coordinates were downloaded from the University of California Santa Cruz (UCSC) genome browser (hg17; http://genome.ucsc.edu/). Genomic regions demonstrating conservation across species and the respective PHAST scores were downloaded from the UCSC genome browser (phastConsElements.txt.gz). Copy number variant (CNV) regions of the genome were obtained from the Database of Genomic Variants (http://projects.tcag.ca/variation/), consisting of 2,714 CNV loci (February 2007). All SNP coordinates are shown with respect to build 35 and dbSNP124.

### Data quality.

To validate the whole-genome genotyping panels, accuracy and completeness of the genotypes generated on both the HumanHap550 and HumanHap650Y panels were measured with respect to call rates, Mendelian inconsistencies, reproducibility, and concordance to HapMap genotype data. These data quality parameters are shown in Table 4.

**Table 4:**
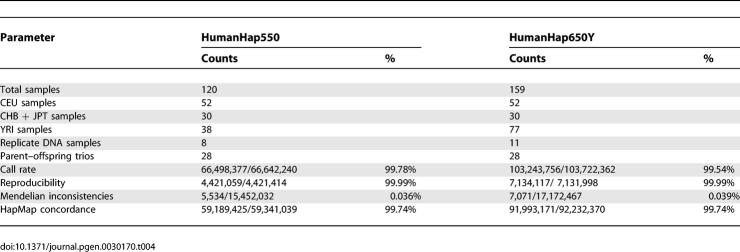
HumanHap550 and HumanHap650Y Data Quality

### Tag SNP selection.

Tag SNPs were by chosen from HapMap data using an algorithm incorporating the LD statistic *r* ^2^ [19]. The genome was divided into 1-Mb nonoverlapping segments, and pairwise *r* ^2^ values were calculated for loci within 200 kb. Approximately 314,000 SNP loci were first selected from the CEU population from the Phase I HapMap data (release 16c). HapMap release 16c had approximately 775,000 SNP loci with MAF ≥ 0.05 in the CEU population. First, tag SNPs were chosen using a strict *r* ^2^ threshold of 0.8. If any SNP in a bin of correlated SNPs was within 10 kb of a RefSeq gene or in an evolutionarily conserved region (ECR), the tag SNP was retained as a “must-keep” SNP. A SNP was defined as being in an ECR if the SNP mapped to one of the phastCons elements with a PHAST score ≥ 50. A second analysis was done using a less-stringent *r* ^2^ threshold of 0.7, choosing additional tag SNPs genome-wide in addition to the “must-keep” tag SNPs selected from the previous analysis. This strategy provided a higher density of tag SNPs within 10 kb of genes or in ECRs.

To construct the HumanHap550, an additional ∼240,000 tag SNPs were selected from the Phase II HapMap data (release 20) and combined with 313,505 HumanHap300 loci. Using the HumanHap300 tag SNP list as “must-haves,” an analysis was conducted using the full release 20 data in the CEU population (>2,100,000 SNPs with MAF ≥ 0.05), prioritizing tag SNP selection for those loci that were polymorphic in all HapMap populations. Again, SNP selection in the CEU population was done choosing a higher density of tag SNPs within 10 kb of RefSeq genes and in ECRs (*r* ^2^ = 0.8 in gene regions/ECRs; *r* ^2^ = 0.7 in rest of the genome). All tag SNPs were retained with the exception of singleton bins (those SNPs not tagging any additional SNPs) not within 10 kb of a gene or in an ECR. An additional tag SNP was selected for those bins with 10 or more loci. After the core set of tag SNPs were determined in the CEU population, additional tag SNPs were included from the Han Chinese/Japanese (CHB + JPT; all bins >2 SNPs at *r* ^2^ = 0.8) and Yoruba populations (YRI; all bins >4 SNPs at *r* ^2^ = 0.7), respectively. Additional content was added to the panel including 7,779 nsSNPs, 177 mitochondrial SNPs (selected from http://www.broad.harvard.edu/mpg/tagger/mito.html [57]), 4,284 SNPs in 495 reported copy number regions of the genome [58–60], and a higher density of tag SNPs in the MHC region. After this final list was selected, any gaps ≥ 100 kb between common SNPs for each population were filled with common SNPs for that particular population. The mean spacing between consecutive common SNPs on autosomal chromosomes is 5.5 kb, 6.5 kb, and 6.3 kb for CEU, CHB + JPT, and YRI, respectively.

To construct the HumanHap650Y, 100,000 additional YRI-specific tag SNPs were added to the 555,532 previously selected SNPs. Using the 555,532 tag SNPs list as “must-haves,” an analysis was conducted using the release 20 data in theYRI population and tag SNPs from the largest bins were selected (bins >2 SNPs, *r* ^2^=0.7). The mean spacing between consecutive common SNPs on autosomal chromosomes is 5.3 kb, 6.2 kb, and 5.4 kb across the genome in the CEU, CHB + JPT, and YRI populations, respectively.

To calculate coverage of HapMap or SeattleSNPs, pairwise *r* ^2^ values were calculated using the expectation algorithm [20] based on the genotypes from HapMap release 21 and the 68 genes resequenced in the PGA samples. Maximum *r* ^2^ values were calculated for each SNP list (HapMap release 20 or 68 PGA genes) with a SNP on either HumanHap550 or HumanHap650Y. All pairwise combinations were considered within 200 kb. For chrX, only female individuals were used; otherwise, all unrelated individuals were used.

### Power to detect risk alleles.

To calculate the power to detect a risk allele with a given risk, risk allele frequency and sample size we generated a series of simulated datasets. For a single sample, we assigned the genotype homozygous for the risk allele and heterozygous or homozygous for the nonrisk allele with Hardy-Weinberg probabilities *p* ^2^, 2*pq*, *q* ^2^ where *p* is the frequency of the risk allele and *q* = 1 − p is the frequency of the nonrisk allele. An individual sample was assigned a disease status with probabilities defined by the disease model and assigned as a case or control accordingly. For example, for an associated risk, λ, under a multiplicative model and a baseline risk, π, an individual has the probability being a case of λ^2^π, λπ and π depending on whether that individual is homozygous for the risk allele, heterozygous, or homozygous for the nonrisk allele, respectively [61]. Using a program written in C, we generated the allele frequencies for 10,000 case-control simulations following the above procedure for a wide range of risk allele frequencies, disease models, and sample sizes. We then calculated the χ^2^ value for the corresponding 2 × 2 table of allele frequencies and estimated the power for each case as the fraction of times that the χ^2^ value exceeded the *p*-value 0.05 after a Bonferroni correction for multiple testing (χ^2^ ≥ 28.5687 for HumanHap550 and χ^2^ ≥ 28.8976 for HumanHap650). This amounted to 188,600,000 simulated case control calculations for each disease model examined (multiplicative and additive).

Using the above power estimates, for each genotyped SNP or SNP tagged at *r* ^2^ = 1, we assigned it a power value according to its minor allele frequency for each disease model and study design (i.e., number of cases and controls). This allows us to do a single power estimation for each of the possible minor allele frequencies (5%–50% in increments of 1%) rather than doing an individual calculation for all possible ∼2.2 million common SNPs. This method works when the SNP is directly genotyped or perfectly correlated with one of the genotyped SNPs. Alternatively, if the risk allele is not directly genotyped but instead, one or several nearby markers are genotyped, then the power is expected to be reduced if none of these genotyped markers are perfectly correlated with the risk allele. The amount that the power is reduced is equivalent to reducing the study sample size by *r* ^2^, where *r* ^2^ is the maximum LD between the genotyped markers and the risk allele [22]. For example, genotyping a SNP that is in LD with a risk allele at *r* ^2^ = 0.5 in 1,000 cases and controls has the same power as directly genotyping the risk allele in 500 cases and controls. In this example, the effective sample size is 500 and is equal to the actual sample size multiplied by *r* ^2^. When the power for the effective sample size was not previously calculated, we linearly interpolated between the power values calculated for sample sizes above and below to estimate the power.

The probability of detecting a SNP in a case control study is the probability that the SNP is a risk allele multiplied by the power to detect it if it is a risk allele [62]. Assuming that a single risk allele is involved in a disease and each SNP is equally likely to be a risk allele, then the total power, *P_T_*, for all SNPs is just the sum of the powers for each SNP and given by the equation:

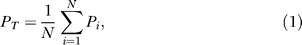
where *P_i_* is the power to detect an association at SNP *i* and *N* is the number of SNPs.

Writing the probability that a SNP is in the HapMap data as *P_H_*, the total power for the entire genome, *P_A_*, can be written as:

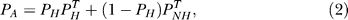
where *P_H_* is the probability that the risk allele is in the HapMap data and (1 − *P_H_*) is the probability that that risk allele is outside of the HapMap data, 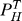 is the power to detect one of the HapMap SNPs, and 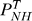 is the power to detect one of the non-HapMap SNPs. It is estimated that *P_H_*, common SNPs represented in the HapMap data, represents 30% of all common SNPs [24], so *P_H_* = 0.30. The power values, 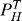 and 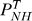 , are taken from the simulated estimates according to the disease model and study design (e.g., Tables S1–S4).

### Power to detect multiple risk alleles.

For a given set of *N* risk alleles of unknown frequency and total powers *P*_0_, *P*_1_,…,*P_N_*, the probability of detecting at least one of these risk alleles—if they are independent of each other—is equal to 1 − (1 − *P*_0_)(1 − *P*_1_),…,(1 − *P_N_*), assuming the risk alleles are independent of one another. While knowing the risks associated with all disease markers is not likely, the lower bound of this estimate can be calculated as 1 − (1 − *P_i_*)*^N^* where *P_i_* is the power for the lowest-powered risk allele and *N* is the number of independent loci. Thus, if the power to detect a single risk allele is 0.5 and there are two risk alleles, then the power to detect one of these risk alleles is 0.75, and if there are three risk alleles, then the power to detect at least one risk allele is 0.875.

## Supporting Information

Figure S1 Total Power to Detect a Single Common Risk Allele in the HapMap Data Using HumanHap550 for CEU (A) and CHB + JPT (B) and HumanHap650Y for YRI (C) in a Study Size of 1,000 Cases and 1,000 Controls under an Additive Disease Model Power is calculated for a 5% false discovery rate after a Bonferroni correction for multiple testing (red line). The solid black line represents the power to detect a risk allele if all the common HapMap SNPs in each respective population are genotyped using the same significance cutoff. Also shown are the powers for different frequency ranges as dashed lines where the lower line indicates the power for risk allele frequencies from 5%–10% and the upper line indicates the power for risk allele frequencies from 40%–50%.
(17 KB PDF)

Figure S2 The Minimum Risk Detectable at 80% Power for Various Sample Sizes (*N* Cases and *N* Controls) under Multiplicative (Red) and Additive (Blue) Models Power is calculated as the ability to detect a single common risk allele in the HapMap data in a whole-genome association study within CHB + JPT (A) and YRI (B) samples.
(17 KB PDF)

Figure S3 Total Power to Detect a Single Common Risk Allele Outside of the HapMap Data Using 1,000 Cases and 1,000 Controls under an Additive Model The power is estimated using the common SNPs in 68 resequenced SeatleSNPs genes that were not characterized in the HapMap Project for (A) 23 CEU samples using HumanHap550 and (B) 24 YRI samples using HumanHap650Y. Power is calculated for a 5% false discovery rate after a Bonferroni correction for multiple testing (red line). The solid black line represents the power to detect a common risk allele in the HapMap data from Figure S1.
(13 KB PDF)

Figure S4 The Minimum Risk Detectable for Common SNPs outside of the HapMap Data at 80% Power for Various Sample Sizes (*N* Cases and *N* Controls) under Multiplicative (Red) and Additive (Blue) Models Power is calculated using the SeattleSNPs data and defined as the ability to detect a single common risk allele outside the HapMap data in a whole-genome association study within either CHB + JPT (A) or YRI (B) samples.
(16 KB PDF)

Table S1 Tajima's *D* statistic on PGA genes (31 KB DOC)

Table S2 Power in CEU Samples for Multiplicative Model (102 KB DOC)

Table S3 Power in CEU Samples for Additive Model (103 KB DOC)

Table S4 Power in YRI Samples for Multiplicative Model (105 KB DOC)

Table S5 Power in YRI Samples for Additive Model (103 KB DOC)

## Abbreviations

CEU: Utah residents with ancestry from Northern and Western Europe
CNV: copy number variant
CHB + JPT: Han Chinese/Japanese in Tokyo, Japan
ECR: evolutionarily conserved region
LD: linkage disequilibrium
MAF: minor allele frequency
PGA: Program for Genomic Applications
YRI: Yoruba in Ibadan, Nigeria
