# Supplementary material for: Power to Detect Risk Alleles Using Genome-Wide Tag SNP Panels
Source: PLoS Genet. 2007 Oct 5;3(10):e170. doi: 10.1371/journal.pgen.0030170 (PMC2000969; doi:10.1371/journal.pgen.0030170)
Supplement: Supplementary file 3 [file pgen.0030170.sg003.pdf]

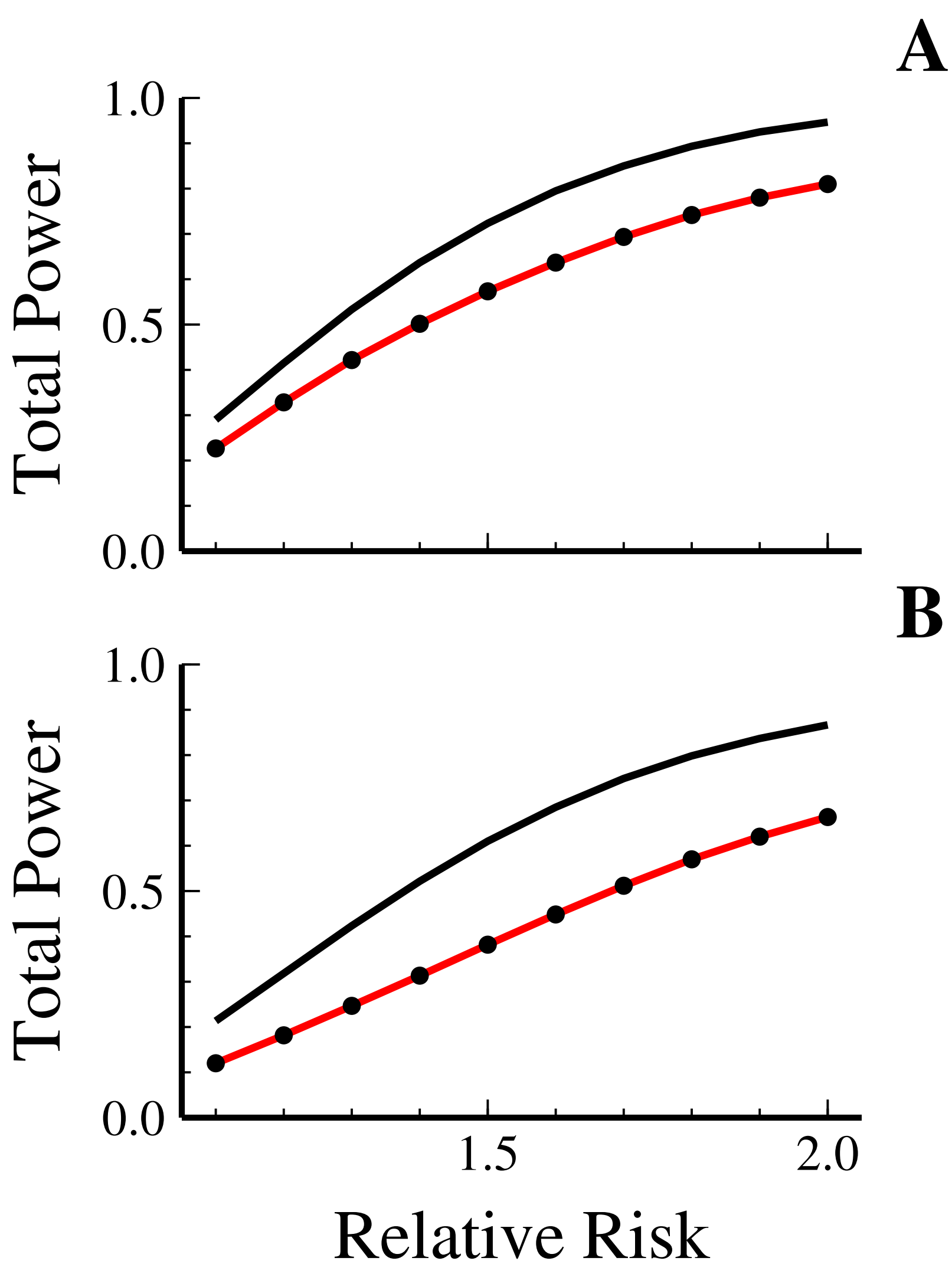
A
B
Total Power
1.0
0.5
0.0
1.5
2.0
Relative Risk
