# Supplementary material for: Power to Detect Risk Alleles Using Genome-Wide Tag SNP Panels
Source: PLoS Genet. 2007 Oct 5;3(10):e170. doi: 10.1371/journal.pgen.0030170 (PMC2000969; doi:10.1371/journal.pgen.0030170)
Supplement: Supplementary file 5 [file pgen.0030170.st001.doc]

**Table S1.** Tajima’s D statistic on PGA genes.

|  | All PGA genes (289) | 68 genes used for coverage and power calculations |
| --- | --- | --- |
| African American samples | -0.51 ± 0.31 | -0.37 ± 0.87 |
| CEPH samples | 0.16 ± 1.04 | 0.24 ± 1.20 |
| All samples | -0.64 ± 0.93 | -0.54 ± 1.00 |
