# Supplementary material for: Power to Detect Risk Alleles Using Genome-Wide Tag SNP Panels
Source: PLoS Genet. 2007 Oct 5;3(10):e170. doi: 10.1371/journal.pgen.0030170 (PMC2000969; doi:10.1371/journal.pgen.0030170)
Supplement: Supplementary file 8 [file pgen.0030170.st004.doc]

**Table S4.** Power in YRI samples for multiplicative model.

|  |  | λ=1.1 |  |  | λ=1.2 |  |  | λ=1.3 |  |  | λ=1.4 |  |  | λ=1.5 |  |
| --- | --- | --- | --- | --- | --- | --- | --- | --- | --- | --- | --- | --- | --- | --- | --- |
| Samples | HM^1^ | SS^2^ | WG^3^ | HM | SS | WG | HM | SS | WG | HM | SS | WG | HM | SS | WG |
| 500 | 0.00 | 0.00 | 0.00 | 0.00 | 0.00 | 0.00 | 0.00 | 0.00 | 0.00 | 0.02 | 0.01 | 0.01 | 0.07 | 0.03 | 0.04 |
| 1000 | 0.00 | 0.00 | 0.00 | 0.00 | 0.00 | 0.00 | 0.03 | 0.02 | 0.02 | 0.17 | 0.09 | 0.12 | 0.39 | 0.22 | 0.27 |
| 2000 | 0.00 | 0.00 | 0.00 | 0.03 | 0.02 | 0.02 | 0.27 | 0.15 | 0.18 | 0.57 | 0.34 | 0.41 | 0.74 | 0.50 | 0.57 |
| 3000 | 0.00 | 0.00 | 0.00 | 0.11 | 0.06 | 0.08 | 0.51 | 0.30 | 0.36 | 0.74 | 0.50 | 0.57 | 0.85 | 0.64 | 0.71 |
| 4000 | 0.00 | 0.00 | 0.00 | 0.24 | 0.13 | 0.17 | 0.65 | 0.41 | 0.48 | 0.83 | 0.61 | 0.67 | 0.91 | 0.73 | 0.78 |
| 5000 | 0.01 | 0.00 | 0.00 | 0.37 | 0.21 | 0.26 | 0.74 | 0.50 | 0.57 | 0.88 | 0.68 | 0.74 | 0.93 | 0.79 | 0.83 |
| 6000 | 0.01 | 0.01 | 0.01 | 0.48 | 0.28 | 0.34 | 0.80 | 0.57 | 0.64 | 0.91 | 0.74 | 0.79 | 0.95 | 0.84 | 0.87 |
| 7000 | 0.02 | 0.01 | 0.02 | 0.56 | 0.34 | 0.41 | 0.84 | 0.62 | 0.69 | 0.93 | 0.78 | 0.82 | 0.96 | 0.87 | 0.89 |
| 8000 | 0.04 | 0.02 | 0.03 | 0.63 | 0.39 | 0.46 | 0.87 | 0.67 | 0.73 | 0.94 | 0.81 | 0.85 | 0.96 | 0.89 | 0.91 |
| 9000 | 0.06 | 0.03 | 0.04 | 0.68 | 0.44 | 0.51 | 0.89 | 0.70 | 0.76 | 0.95 | 0.84 | 0.87 | 0.97 | 0.91 | 0.93 |
| 10000 | 0.08 | 0.04 | 0.06 | 0.72 | 0.48 | 0.55 | 0.91 | 0.73 | 0.78 | 0.95 | 0.86 | 0.89 | 0.97 | 0.93 | 0.94 |

|  |  | λ=1.6 |  |  | λ=1.7 |  |  | λ=1.8 |  |  | λ=1.9 |  |  | λ=2.0 |  |
| --- | --- | --- | --- | --- | --- | --- | --- | --- | --- | --- | --- | --- | --- | --- | --- |
| Samples | HM^1^ | SS^2^ | WG^3^ | HM | SS | WG | HM | SS | WG | HM | SS | WG | HM | SS | WG |
| 500 | 0.17 | 0.09 | 0.11 | 0.30 | 0.17 | 0.21 | 0.44 | 0.25 | 0.31 | 0.55 | 0.33 | 0.40 | 0.64 | 0.40 | 0.47 |
| 1000 | 0.57 | 0.35 | 0.41 | 0.69 | 0.45 | 0.52 | 0.77 | 0.54 | 0.61 | 0.83 | 0.61 | 0.67 | 0.87 | 0.66 | 0.72 |
| 2000 | 0.83 | 0.61 | 0.68 | 0.89 | 0.70 | 0.76 | 0.92 | 0.76 | 0.81 | 0.94 | 0.81 | 0.85 | 0.95 | 0.85 | 0.88 |
| 3000 | 0.91 | 0.74 | 0.79 | 0.94 | 0.81 | 0.85 | 0.95 | 0.86 | 0.89 | 0.97 | 0.90 | 0.92 | 0.97 | 0.92 | 0.94 |
| 4000 | 0.94 | 0.82 | 0.85 | 0.96 | 0.87 | 0.90 | 0.97 | 0.91 | 0.93 | 0.98 | 0.94 | 0.95 | 0.98 | 0.96 | 0.96 |
| 5000 | 0.96 | 0.86 | 0.89 | 0.97 | 0.91 | 0.93 | 0.98 | 0.94 | 0.95 | 0.98 | 0.96 | 0.97 | 0.99 | 0.97 | 0.98 |
| 6000 | 0.97 | 0.90 | 0.92 | 0.98 | 0.94 | 0.95 | 0.98 | 0.96 | 0.97 | 0.99 | 0.97 | 0.98 | 0.99 | 0.98 | 0.98 |
| 7000 | 0.97 | 0.92 | 0.94 | 0.98 | 0.95 | 0.96 | 0.99 | 0.97 | 0.98 | 0.99 | 0.98 | 0.98 | 0.99 | 0.98 | 0.99 |
| 8000 | 0.98 | 0.94 | 0.95 | 0.99 | 0.96 | 0.97 | 0.99 | 0.98 | 0.98 | 0.99 | 0.98 | 0.98 | 1.00 | 0.98 | 0.99 |
| 9000 | 0.98 | 0.95 | 0.96 | 0.99 | 0.97 | 0.98 | 0.99 | 0.98 | 0.98 | 1.00 | 0.98 | 0.99 | 1.00 | 0.98 | 0.99 |
| 10000 | 0.99 | 0.96 | 0.97 | 0.99 | 0.98 | 0.98 | 0.99 | 0.98 | 0.99 | 1.00 | 0.98 | 0.99 | 1.00 | 0.98 | 0.99 |

^1^HapMap

^2^SeattleSNPs

^3^Whole-Genome
